# The Effect of Alzheimer’s Disease-Associated Genetic Variants on Longevity

**DOI:** 10.3389/fgene.2021.748781

**Published:** 2021-12-21

**Authors:** Niccolò Tesi, Marc Hulsman, Sven J. van der Lee, Iris E. Jansen, Najada Stringa, Natasja M. van Schoor, Philip Scheltens, Wiesje M. van der Flier, Martijn Huisman, Marcel J. T. Reinders, Henne Holstege

**Affiliations:** ^1^ Section Genomics of Neurodegenerative Diseases and Aging, Department of Clinical Genetics, Vrije Universiteit Amsterdam, Amsterdam, Netherlands; ^2^ Alzheimer Centre, Department of Neurology, Amsterdam Neuroscience, Vrije Universiteit Amsterdam, Amsterdam, Netherlands; ^3^ Delft Bioinformatics Lab, Delft University of Technology, Delft, Netherlands; ^4^ Department of Complex Trait Genetics, Center for Neurogenomics and Cognitive Research, VU, Amsterdam, Netherlands; ^5^ Department of Epidemiology and Data Sciences, Vrije Universiteit Amsterdam, Amsterdam, Netherlands

**Keywords:** cognitively healthy, centenarians, aging, alzheimer’s disease, effect on aging, protective variants

## Abstract

Human longevity is influenced by the genetic risk of age-related diseases. As Alzheimer’s disease (AD) represents a common condition at old age, an interplay between genetic factors affecting AD and longevity is expected. We explored this interplay by studying the prevalence of AD-associated single-nucleotide-polymorphisms (SNPs) in cognitively healthy centenarians, and replicated findings in a parental-longevity GWAS. We found that 28/38 SNPs that increased AD-risk also associated with lower odds of longevity. For each SNP, we express the imbalance between AD- and longevity-risk as an effect-size distribution. Based on these distributions, we grouped the SNPs in three groups: 17 SNPs increased AD-risk more than they decreased longevity-risk, and were enriched for *β*-amyloid metabolism and immune signaling; 11 variants reported a larger longevity-effect compared to their AD-effect, were enriched for endocytosis/immune-signaling, and were previously associated with other age-related diseases. Unexpectedly, 10 variants associated with an increased risk of AD and higher odds of longevity. Altogether, we show that different AD-associated SNPs have different effects on longevity, including SNPs that may confer general neuro-protective functions against AD and other age-related diseases.

## Introduction

The human lifespan is determined by a beneficial combination of environmental and genetic factors (Partridge et al., 2018; Melzer et al., 2019). Long-lived individuals tend to cluster in families, suggesting that genetic factors play a considerable role (Perls et al., 2002; Caselli et al., 2006), however, the research of genetic variants that influence human lifespan has yielded contrasting results: only the association of the *APOE* alleles and few additional variants (in/near *CDKN2B* and *ABO*) consistently replicated across studies (Deelen et al., 2019; Timmers et al., 2019). While the replication rate in independent studies is low, a large collection of genetic variants has been associated with longevity through genome-wide association studies (GWAS) in different studies and populations (Deelen et al., 2019; Timmers et al., 2019). The majority of these variants was previously associated with other age-related conditions, including cardiovascular disease, autoimmune and neurological disorders, suggesting that the genetics underlying human longevity depends on a lower genetic risk for age-related diseases (Deelen et al., 2019; Melzer et al., 2019; Timmers et al., 2019).

Of all age-related diseases, late-onset Alzheimer’s disease (AD) is the most common type of dementia and one of the most prevalent causes of death at old age (Alzheimer’s Association, 2012). The largest risk factor for AD is aging: at 100 years of age, the disease’s incidence is about 40% per year (Corrada et al., 2010). Genetic factors play a significant role in AD as heritability was estimated to range 60–80% (Gatz et al., 2006): the strongest common genetic risk factor for AD is the *APOE*-*ε4* allele, and large collaborative GWAS have identified 41 additional common variants associated with a slight modification of the risk of AD (Sims et al., 2017; Jansen et al., 2019; Kunkle et al., 2019; de Rojas et al., 2021). Despite high incidence rates of AD at very old ages, AD is not an inevitable consequence of aging, as demonstrated by individuals who surpass the age of 100 years with high levels of cognitive functioning (Holstege et al., 2018).

As AD-associated variants increase the risk of AD, leading to earlier death, a negative effect on longevity for these variants should be expected. However, apart from *APOE* alleles, genetic variants that influence the risk of AD were not found to affect the human lifespan in previous GWAS. In fact, often we assume that AD-associated variants affect AD only, but this may still not hold true. For example, at the molecular level, there may be other age-related traits that share (part of) the biological pathways underlying AD. Nevertheless, for an AD-associated variant that affects AD only, the relative effect on longevity should be proportional to the corresponding effect on AD, albeit in a different direction. This means that if a variant increases the risk of AD 2-fold, then carriers will have twice as much AD-related mortality as non-carriers, and as a consequence, they will have twice as little chance to age into a cognitively healthy centenarian. However, in case a genetic variant is protective against multiple conditions, it might be expected that the overall effect on longevity results larger than the absolute effect on AD alone.

We have previously shown that cognitively healthy centenarians are depleted with genetic variants that increased the risk of AD compared to a general population. Yet, the extent of depletion was variant specific, suggesting that a subset of AD-variants may be specifically beneficial to reach extremely old ages in good cognitive health (Tesi et al., 2019; Tesi et al., 2020). In addition, the extent to which AD-associated variants affect other age-related diseases is mostly unknown (Ponomareva et al., 2013). Using the notion of effect-size proportionality, we set out to investigate the relationship between AD- and longevity-risk for genetic variants associated with AD.

## Methods

### Populations and Selection of Genetic Variants

We included *N* = 358 centenarians from the 100-plus Study cohort, which comprises Dutch-speaking individuals aged 100 years or older who self-report to be cognitively healthy, which is confirmed by a proxy (Holstege et al., 2018). As population controls, we used population-matched, cognitively healthy individuals from five studies: 1) the Longitudinal Aging Study of Amsterdam (LASA, *N* = 1,779), (Huisman et al., 2011; Hoogendijk et al., 2016), 2) the memory clinic of the Alzheimer center Amsterdam and SCIENCe project (*N* = 1,206), (Slot et al., 2018; van der Flier and Scheltens, 2018), 3) the Netherlands Brain Bank (*N* = 40), (Rademaker et al., 2018), 4) the twin study of Amsterdam (*N* = 201) (Willemsen et al., 2010) and 5) the 100-plus Study (partners of centenarian’s children, N = 86). (Holstege et al., 2018). see *5.1 Supplementary Methods: Populations* for a detailed description of these cohorts. Throughout the manuscript, we will refer to the union of the individuals from these five studies as population subjects. The Medical Ethics Committee of the Amsterdam UMC (METC) approved all studies. All participants and/or their legal representatives provided written informed consent for participation in clinical and genetic studies.

Genetic variants in our populations were determined by standard genotyping and imputation methods. All samples were genotyped using the same commercial kit. After establishing quality control of the genetic data (see *5.2 Supplementary Methods: Quality control*), 2,905 population subjects and 343 cognitively healthy centenarians were left for the analyses (*Table 1*). We then selected 41 variants representing the current genetic landscape of AD (Supplementary *Table S1*) (de Rojas et al., 2021). We restricted our analysis to high-quality variants with a minor allele frequency >1% in our cohorts, which led to the exclusion of 3/41 variants (rare variants in the *TREM2* gene *rs143332484* and *rs75932628* and *ABI3* gene *rs616338*), leaving 38 variants for the analyses.

**TABLE 1:** Population characteristics.

	Population controls	Cognitively healthy centenarians
**Number of individuals**	2,905	343
**Females (%)**	1,400 (48.2)	246 (71.7)
**Age (SD)** **^a^**	68.3 (11.5)	101.4 (1.8)
***ApoE ε4* (%)**	1,012 (17.38)	48 (7.15)
***ApoE ε2* (%)**	523 (9.00)	91 (13.26)

a Age at study inclusion; SD, standard deviation; *ApoE*, Apolipoprotein E allele count for ε4 and ε2, and relative allele frequency in population controls and cognitively healthy centenarians. Reference to the cohorts reported in this table are: (Willemsen et al., 2010; Huisman et al., 2011; Holstege et al., 2018; Rademaker et al., 2018; Slot et al., 2018; van der Flier and Scheltens, 2018).

### Alzheimer’s Disease and Longevity Variant Effect Sizes

We first retrieved the effect-size on AD ( $E_{AD}^{k}$ ) for each variant, *k*, from one of the largest GWAS of AD (de Rojas et al., 2021) To estimate a confidence interval, we sampled (*S* = 10,000) from the published effect-sizes (log of odds ratios) and their respective standard errors. To calculate the effect-size on longevity ( $E_{LGV}^{k}$ ) for the same variants, we used a logistic regression model with cognitive healthy centenarians as cases and population subjects as controls while adjusting for population stratification (PC 1–5). The number of principal components to include as covariates was arbitrarily chosen, however, as all individuals were population-matched, we expected these components to correct all major population effects. The resulting *p*-values were corrected for multiple comparisons (False Discovery Rate, FDR). To calculate the confidence interval, we repeated this procedure for bootstraps (*B* = 10,000) of the data. For convenience, variant effect-sizes on AD and longevity were calculated with respect to the allele that increases the risk of AD, such that $E_{AD}^{k}>0$ by definition. Given a variant *k,* with a relative effect-size on AD ( $E_{AD}^{k}$ ) and on longevity ( $E_{LGV}^{k}$ ), we defined that the variant has an *expected direction* if the variant increases the risk of AD, *i.e.,* $E_{AD}^{k}>0$ , and decreases the risk of longevity, *i.e.,* $E_{LGV}^{k}<0$ . Inversely, we define that the longevity effect has an *unexpected direction* if the allele that increased AD risk also increased the risk of longevity, *i.e.,* $E_{AD}^{k}>0$ and $E_{LGV}^{k}>0$ . The probability of observing an *expected direction* was considered a Bernoulli variable with *p* = 0.5 (*i.e.,* equal chance of having an *expected/unexpected* direction), thus the number of variants with an *expected direction* follows a binomial distribution.

### Imbalance of Variant Effect Direction

We represented each variant as a data point whose coordinates are defined by the variant’s effect on AD ( $E_{AD}^{k}$ , on the *y*-axis) and its effect on longevity ( $E_{LGV}^{k}$ , on the *x*-axis). See Supplementary *Figure S1* for an example. For each variant, we then calculated the normalized angle, $\alpha_{k}$ , of the vector representing the data point with the *x*-axis: $\alpha_{k}=\frac{atan2\left(E_{AD}^{k},\;E_{LGV}^{k}\right)}{\pi /2}+1$ , with $\alpha_{k}\in \left[-1;1\right]$ . This normalized angle relates to the imbalance between the risk of AD and the risk of longevity. That is, for $\alpha_{k}<0$ the variant has an expected direction, while for $\alpha_{k}>0$ the variant has an unexpected direction (Supplementary *Figure S1*). As the effect-sizes are sample estimates, we took their confidence interval into account to create, for each variant, a distribution of the imbalance in the effect direction ( IED ). Hereto, we assumed a Gaussian density for both $E_{AD}^{k}$ and $E_{LGV}^{k}$ , centered around ${\bar{E}}_{AD}^{k}$ and ${\bar{E}}_{LGV}^{k}$ and with a variance equal to the estimated confidence interval for both effect-sizes, respectively. We sampled 10,000 times from these distributions and calculated the corresponding imbalance ( $\alpha_{k})$ , to get a (non-Gaussian) distribution of the IED  for each variant, $IED_{k}$ . To group variants with similar patterns of their IED distributions, we ordered the IED by their median value ${\tilde{IED}}_{k}$ , and defined a group of variants in which the effect-sizes were in the expected direction ( ${\tilde{IED}}_{k}\leq 0$ ), which we subsequently split in those that have 1) a larger effect on longevity as compared to the effect on AD ( ${\tilde{IED}}_{k}\leq -1/2$ , *Longevity-group*), and those that have 2) a larger effect on AD as compared to the effect on longevity ( $-1/2<{\tilde{IED}}_{k}\leq 0$ , *AD-group*). We defined a third group of variants that have an effect in the unexpected direction ( ${\tilde{IED}}_{k}>0$ , *Unex*-*group*). These cut-off values were not arbitrarily chosen, instead, they represent the point at which the effect on AD equals the (negative) effect on longevity ( $IED_{k}=-1/2$ ) and the point at which no effect on longevity is observed ( $IED_{k}=0$ ).

### Replication of Findings in Large Genome-Wide Association Studies Cohorts

To find additional evidence for our findings, we inspected the association statistics of the 38 AD-associated variants in the largest GWAS on parental longevity. (Timmers et al., 2019). Briefly, in this study offspring’s genotypes were used to model parental age at death. We did not use a case-control GWAS of longevity as the most recent included our cohort, thus the resulting associations would be biased. (Deelen et al., 2019). In this dataset, we looked at the significance of association with longevity of the 38 variants (*p*-values were corrected with FDR) and their direction of effect. Finally, we tested the consistency in the expected/unexpected directions between our study and the GWAS on parental longevity using binomial tests.

### Linking Variants With Functional Clusters

To investigate each variant’s functional consequences, we calculated the variant-pathway mapping, which indicates the degree of involvement of each genetic variant in AD-associated pathways (Supplementary *Figure S2*). see *5.3 Supplementary Methods: variant-pathway mapping* for a detailed explanation of our approach. Briefly, the variant-pathway mapping depends on 1) the number of genes each variant was associated with and 2) the biological pathways each gene was associated with. We calculated the variant-pathway mapping for all 38 AD-associated variants. Finally, we compared the variant-pathway mapping within each group of variants defined based on the IEDs (Longevity-, AD- and Unex-groups) using Wilcoxon sum rank tests and correcting *p*-values using FDR: this was indicative of whether a group of variants was enriched for a specific functional cluster (Supplementary *Figure S2*).

### Cell-Type Annotation at The Level of Each Cluster

To further explore the biological basis of the different groups of variants (Longevity-, AD- and Unex-groups), we calculated the degree of enrichment of each group for specific brain cell-types (see *5.4 Supplementary Methods: cell-type annotation* for a detailed description). This annotation depends on the number of genes each variant was associated with, and the expression of these genes in the different brain cell-types, *i.e.,* astrocytes, oligodendrocytes, microglia, endothelial cells, and neurons. We finally compared the cell-specific annotations within each group of variants (Longevity-, AD- and Unex-groups) using Wilcoxon sum rank tests and correcting *p*-values using FDR, which indicated whether a group of variants was enriched for specific brain cell-types (Supplementary *Figure S2*).

### Implementation

Quality control of genotype data, population stratification analysis and relatedness analysis were performed with PLINK (v2.0 and v1.9). All subsequent analyses were performed with R (v3.6.3), Bash, and Python (v3.6) scripts. All scripts are freely available at https://github.com/TesiNicco/Disentangle_AD_Age. Variant-gene annotation and gene-set enrichment analyses were performed through the web-server that is freely accessible at https://snpxplorer.net. (Tesi et al., 2021).

## Results

### Alzheimer’s Disease-Associated Variants Also Associate With Longevity

We explored the association with longevity of 38 genetic variants previously associated with AD from GWAS (Supplementary *Table S1*). We tested these variants in 343 centenarians who self-reported to be cognitively healthy (mean age at inclusion 101.4 ± 1.3, 74.7% females), as opposed to 2,905 population subjects (mean age at inclusion 68.3 ± 11.5, 50.7% females). We found a significant association with longevity for two variants after multiple testing correction (FDR<5%, variants in the *APOE* gene; *rs429358* and *rs7412*, Supplementary *Table S2*). We compared the direction of effect on longevity with that on AD as found in literature: of the 38 variants, 28 showed an association in the expected direction, *i.e.,* alleles that increased AD risk were associated with lower odds of longevity, and this was significantly more than expected by chance (*p* = 0.005 including *APOE* variants, *p* = 0.01 excluding *APOE* variants, see *2.2 Methods*).

### Distributions of the Imbalance in the Effect Direction ( IED )

**FIGURE 1:**
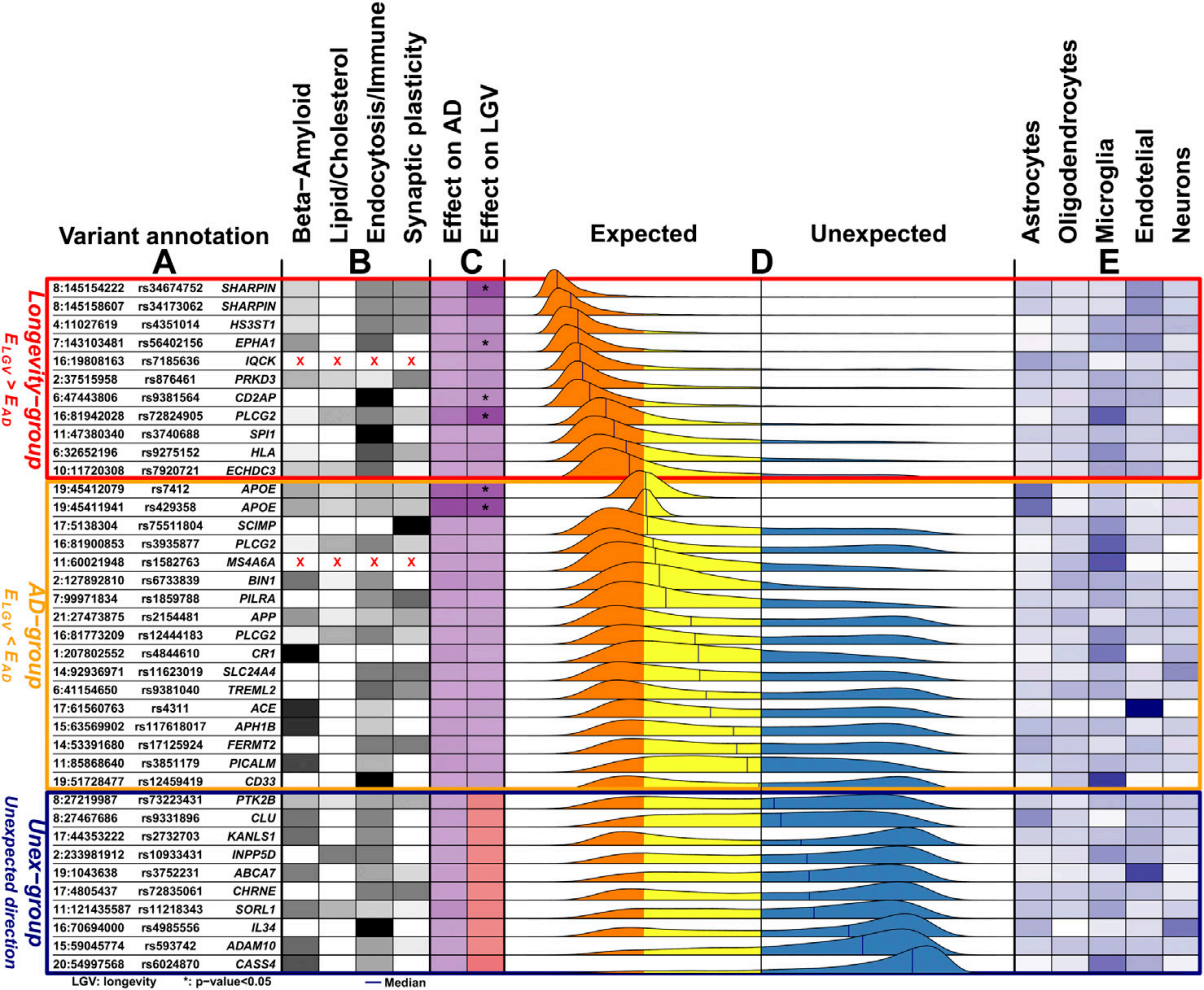
Overview of the 38 genetic variants associated with Alzheimer’s disease. **(A)** The genomic position of the variants (chromosome: position), variant identifier, and closest gene. Genomic positions are with respect to GRCh37 (hg19). **(B)** The variant-pathway mapping score of association with the four functional clusters (darker colors representing stronger associations). Variants annotated with red crosses could not be annotated to any one of the functional clusters as no biological processes are associated with the related genes. **(C)** The effect size on AD (from literature) and the observed effect size on longevity (LGV) for each variant (darker color indicating stronger effect). The same color indicates *expected direction* (*i.e.,* increased risk of AD and decreased chance of longevity), while different colors, visible in the *Unex-group* of variants, indicates *unexpected direction*. For the longevity effects, we also annotate variants for which we observed a significant association (unadjusted *p-value* < 0.05). **(D)** The distribution of the imbalance direction of variant effect ( IED ) in AD-risk as compared to cognitive health aging (see *2.3 Methods* for details). The *Longevity-, AD-* and *Unex-groups* were derived based on the median value of the IED . The median value is reported for each IED as a blue vertical line. **E.** Average gene expression of the genes associated with the variant in five different brain cell-types (the darker, the higher the expression).

To study the relationship between the effect on AD and longevity for all 38 AD-associated variants in more detail, we created distributions of the imbalance in the variant effect direction ( IED ): *Figure 1*. The IED of a variant indicates 1) whether the effects on AD and longevity are in the expected or unexpected direction, and 2) how the effects on AD and longevity relate to each other. For example, the two variants *rs7412* and *rs429358* in *APOE* gene significantly associated with longevity in the expected direction and thus had tight confidence intervals. The resulting IED relied completely in the expected direction side (*Figure 1*). In addition, the effect on AD was larger than that on longevity, causing the IED to slightly skew towards the AD-side (*Figure 1*). However, as the association of a variant with longevity became less strong (thus with larger confidence intervals) or was in the unexpected direction, the fraction of data points in the unexpected direction side increased. For example, for the intergenic variant *rs6733839* close to *BIN1* gene, we observed a larger effect on AD compared to cognitively healthy aging ( $E_{AD}^{BIN1}$ = 0.17, SE = 0.01 and $E_{LGV}^{BIN1}$ = –0.14, SE = 0.08, *p* = 0.09), yet in the expected direction. The resulting IED is skewed towards the AD side (*Figure 1*), and, due to large confidence intervals on longevity, we observed data points in the unexpected direction. Finally, variant *rs593742* near *ADAM10* gene ( $E_{AD}^{ADAM10}$ = 0.08, SE = 0.01 and $E_{LGV}^{ADAM10}$ = 0.06, SE = 0.09, *p* = 0.49) associated with higher odds of both AD and longevity (unexpected direction of effect), with a resulting IED largely on the unexpected side with fewer data points on the expected direction (due to large confidence intervals).

### Grouping Variants Based on IED Distributions

Based on the median value of each IED distributions, ${\tilde{IED}}_{k},$ we grouped the variants into 1) a Longevity-group (variants with a ${\tilde{IED}}_{k}$ skewed towards the longevity*-*end of the spectrum), 2) an AD-group (variants with a ${\tilde{IED}}_{k}$ skewed towards the *AD*-end of the spectrum), and 3) an Unex-group (variants with a ${\tilde{IED}}_{k}$ in the unexpected direction). The *AD-*group included 17 variants (*APOE* (1), *APOE* (2), *SCIMP*, *PLCG2* (1), *MS4A6A*, *BIN1, PILRA, APP, PLCG2* (2), *CR1, SLC24A4, TREML2, ACE, APH1B, FERMT2, PICALM, CD33*) and the Longevity*-*group included 11 variants (*SHARPIN* (1), *SHARPIN* (2), *HS3ST1, EPAH1, IQCK, PRKD3, CD2AP, PLCG2* (3), *SPI1, HLA, ECHDC3*), such that the effect of 28/38 (74%) of all variants was in the expected direction. The effect of 10 variants was in the unexpected direction, the Unex-group: (*PTK2B, CLU, KANSL1, INPP5D, ABCA7, CHRNE, SORL1, IL34, ADAM10, CASS4*) (*Figure 1*).

### Alzheimer’s Disease-Associated Variants in Large Genome-Wide Association Studies of Longevity

**FIGURE 2:**
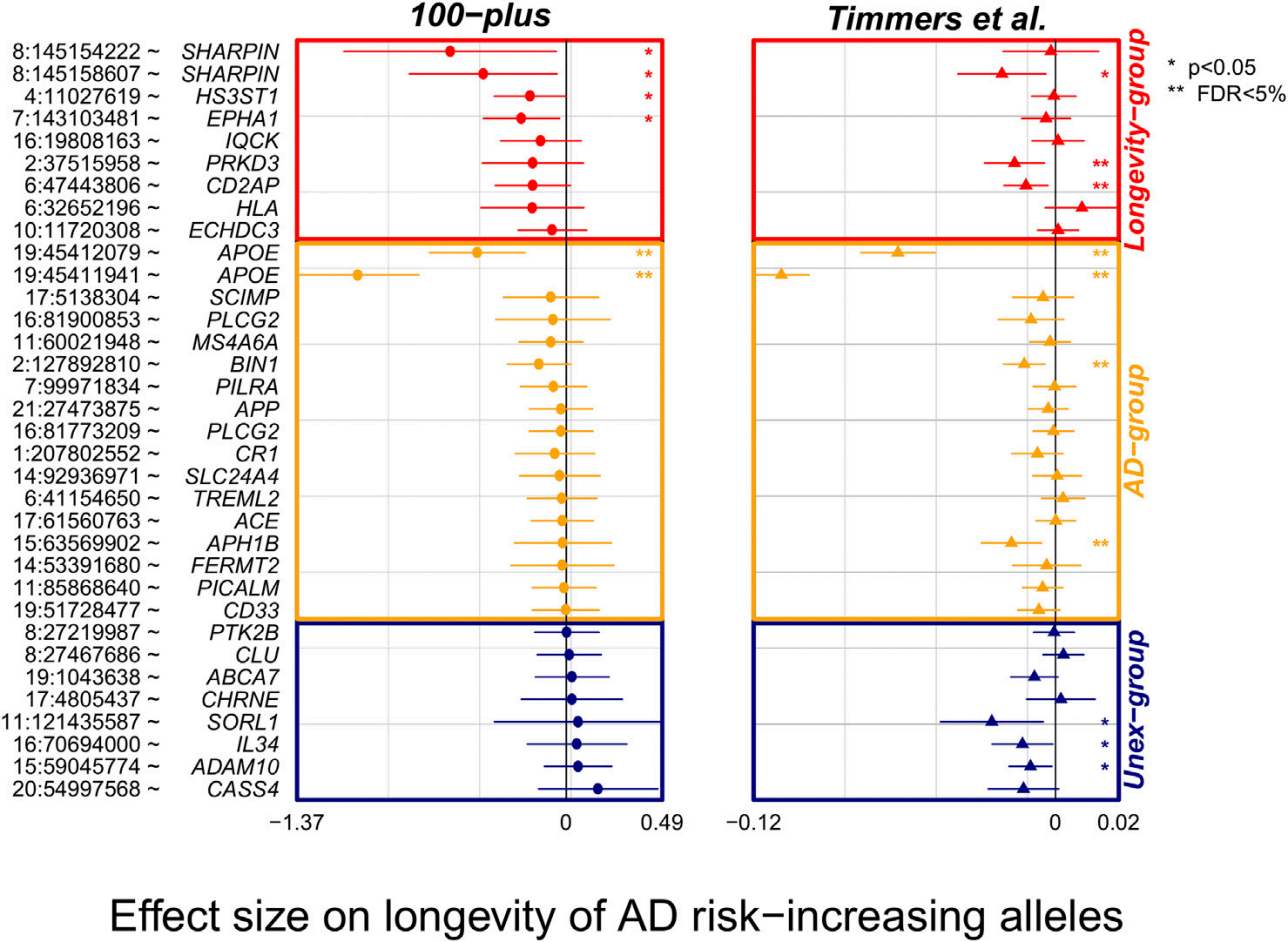
Forest plot of association statistics of AD-variants in our study and the largest GWAS of parental longevity. The plot shows the association of AD-variants in our study and the largest by-proxy GWAS on parental longevity (Timmers et al., 2019). The association statistics of 34/38 variants were available from publicly available summary statistics of Timmers et al. study. Plotted effect-sizes are with respect to the AD-risk increasing allele. Thus, an expected direction of effect is shown for variants with a negative estimate. Nominally significant associations with AD (*p <* 0.05) are annotated with an asterisk (*), and significant associations after FDR correction are annotated with two asterisks (**).

To find additional evidence for longevity associations, we inspected the effect of AD-associated variants in the largest GWAS of parental longevity. (Timmers et al., 2019). Of the 38 AD-associated variants, association statistics were available for 34 of the variants (missing from Longevity-group: *rs72824905* and *rs3740688;* missing from Unex-group*: rs2732703* and *rs10933431*). Overall, 21/26 (81%) of the variants in the expected direction in our study (of which 6/9 variants in Longevity- and 15/17 variants in the AD-group), were also in the expected direction in the independent parental longevity dataset. Variants in the expected direction in the first analysis were significantly more likely to be in the expected direction in the replication analysis (*p* = 0.01, based on a binomial test, *Figure 2*). Six AD-associated variants reached significance in the parental-longevity GWAS after correcting for multiple comparisons (FDR<5%): variants in the *APOE* gene (*rs429358* and *rs7412*) and those in/near *PRKD3 (rs8764613)*, *CD2AP (rs9381564), APH1B* (*rs117618,017*) and *BIN1* (*rs6733839*). Of these, variants in/near *PRKD3* and *CD2AP* belonged to the Longevity-group in our analysis. Conversely, only 2/8 (25%) variants that we observed in the unexpected direction in our study were also in the unexpected direction in the parental-longevity GWAS, such that these variants were *not* more likely to be in the unexpected direction (*p =* 0.29, based on a binomial test, *Figure 2*).

### Functional Characterization of Variants

The 38 AD-associated variants included coding variants (*N* = 10), intronic variants (*N* = 20), and intergenic variants (*N* = 8) (Supplementary *Table S3*). 12/28 of the intronic/intergenic variants had eQTL associations. In total, the 38 variants mapped to 68 unique genes, with most variants mapping to one gene (*N* = 21) and fewer mapping to 2 genes (*N* = 10), 3 genes (*N* = 2), 4 genes (*N* = 1), 5 genes (*N* = 2), 6 and 7 genes (*N* = 1, respectively) (Supplementary *Figure S3* and Supplementary *Table S3*).

We performed gene-set enrichment analysis using a sampling-based approach to explore the biological processes enriched in the 68 genes associated with AD-variants (see *2.4 Methods* and Supplementary *Figure S2*). We found 115 significantly enriched biological processes after correction for multiple tests (FDR<5%, Supplementary *Table S4*). After clustering these terms based on their semantic similarity, we found four main clusters of biological processes: 1) β -amyloid metabolism, 2) lipid/cholesterol metabolism, 3) endocytosis/immune signaling and 4) synaptic plasticity (*Figure 1* *,* *Supplementary Figure S4* and Supplementary *Table S5*).

**FIGURE 3:**
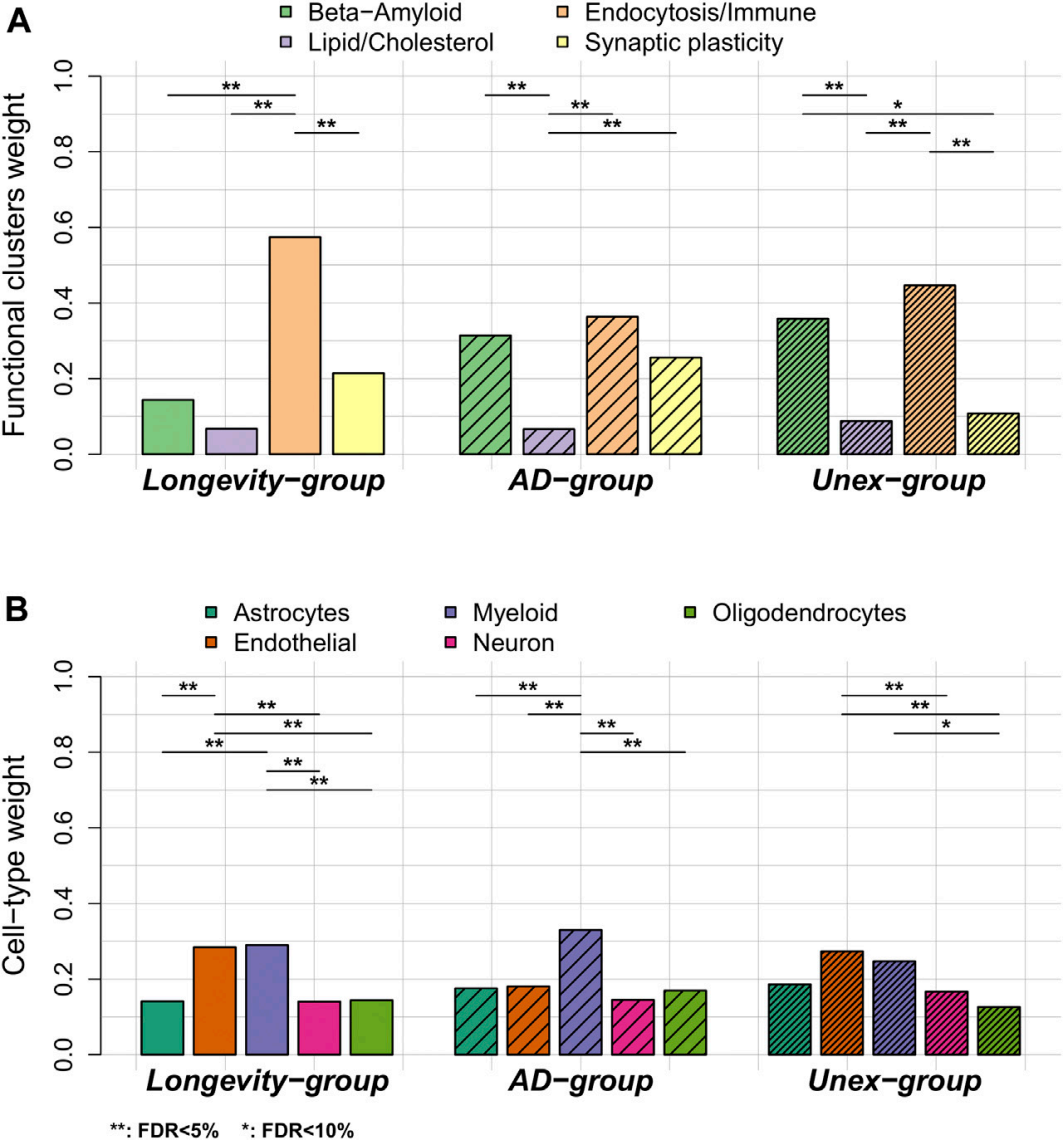
Comparison of functional annotation and cell-type annotation within the *Longevity-, AD-* and *Unex-groups*. **(A)** The weights of the 4 functional clusters within the *Longevity-, AD-* and *Unex-groups*. **(B)** The weights of the different cell-types in the brain, per group. Differences in functional weights and cell-type weights within each group were calculated using Wilcoxon sum rank tests. The resulting *p*-values were FDR-corrected.

• Next, we calculated the variant-pathway mapping score (see *2.4 Methods* and Supplementary *Figure S2*)*,* which indicates how well a variant is associated with each of the 4 functional clusters. In total, we calculated the variant-pathway mapping for 30 variants and we imputed the annotation of 6 variants (Supplementary *Table S5*), while 2 variants could not be annotated (variants *rs7185636* and *rs1582763* in/near *IQCK* and *MS4A6A* genes), because the associated genes were not annotated with any biological process function (Supplementary *Table S5*). Finally, we tested whether the Longevity-, AD- and Unex-groups were enriched at the functional level by comparing the distribution of variant-pathway mapping within each group (see *2.4 Methods* *,* *Figure 3* *,* and Supplementary *Figure S2*). The Longevity-group was significantly enriched for the endocytosis/immune signaling functional cluster; the AD-group for the endocytosis/immune signaling, β -amyloid metabolism and to a smaller extent for the synaptic plasticity functional clusters; the Unex-group was mainly enriched for the endocytosis and β -amyloid metabolism functional clusters.

### Expression of Alzheimer’s Disease-Associated Genes in Brain Cell-types

We explored whether specific brain cell types, *i.e.,* astrocytes, oligodendrocytes, microglia, endothelial cells and neurons, were enriched within each group of variants (see *2.5 Methods* *,* *Supplementary Tables S5,* S6). *Figure 1* shows the collapsed cell-type expression for all 38 AD-associated variants. We then tested the enrichment for cell-type expression within the Longevity-, AD- and Unex-groups. The Longevity*-*group was significantly enriched for myeloid and endothelial cells, the *AD-*group for myeloid cells, while the Unex*-*group was significantly enriched for endothelial cells (*Figure 3*).

## Discussion

### Summary of the Findings

We studied the effect on longevity of 38 genetic variants previously associated with AD through GWAS (de Rojas et al., 2021). We found that a majority of 74% of the alleles that increase the risk of AD is associated with lower odds of becoming a centenarian (expected direction). Overall, most variants (*N* = 17) had a larger effect on AD than on longevity: these variants were associated with β -amyloid metabolism and endocytosis/immune signaling, and were primarily expressed in microglia. A subset of variants (*N* = 11) had a larger effect on longevity than their effect on AD. These variants were associated mostly with endocytosis and immune signaling, and they were expressed in microglia and endothelial cells. These variant-effects were confirmed for 81% of the alleles in an independent dataset, the largest GWAS on parental longevity. In contrast, 26% of the variants increased both the risk of developing AD *and* the risk of becoming a centenarian (*N* = 10), (unexpected direction). These unexpected effects could only be replicated for 2 of the variants in the independent dataset, and none of the SNPs in this group was significantly associated with longevity. Therefore, a larger sample size and/or replication in additional independent cohorts of long-lived individuals would be required to confirm these associations. Together, our findings suggest that a subset of variants associated with AD-risk may also affect longevity, for example through their effect on other age-related diseases.

### Alzheimer’s Disease-Associated Variants and Their Effect on Healthy Aging

A single study previously explored the extent to which 10 AD-associated variants affect longevity: apart from *APOE* locus, none of the other 10 tested AD-associated variants significantly associated with longevity (Shi et al., 2012). In addition to *APOE,* four variants showed a negative effect on longevity while increasing AD-risk (in/near *ABCA7, EPHA1, CD2AP,* and *CLU*). In agreement with these findings, we also found that only the *APOE* variants significantly associated with longevity, and variants in/near *EPHA1* and *CD2AP* belong to the Longevity-group. However, in our study, we found that most alleles associated with an increased risk of AD associated with a decreased chance of longevity. The inability to observe such an inverse relationship between variant effects on AD and longevity in the previous study may be explained by the relatively small sample sizes, combined with a low number of (well-established) AD variants analyzed (*N* = 10). In our study, groups sizes were also relatively small, but the centenarians had a relatively high level of cognitive health, which might have contributed to an increased effect size of AD-associated genetic variants in our comparison (Tesi et al., 2019; Tesi et al., 2020).

### Different Trajectories of Effect of Alzheimer’s Disease-Associated Variants on Longevity

#### Variants With a Larger Effect on Alzheimer’s Disease Than Longevity

For most variants with effects in the expected direction, the risk-increasing effect on AD was more extensive than the negative effect on survival/longevity. These variants, which include both *APOE* alleles, might negatively affect lifespan because carriers are removed from the population with increasing age due to AD-associated mortality. For the *APOE* variants specifically, the distribution of the imbalance in the effect directions ( IED ) suggests a nearly similar magnitude of the increased risk of AD and decreased risk of longevity ( ${\tilde{IED}}_{k}\approx -1/2$ ). This explains why multiple previous studies have associated *APOE* variants with longevity. In our cohort of centenarians, the frequency of the *ε4* allele, which increases AD-risk, is half of that of the population controls (8% *vs* 16%, respectively). In comparison, the frequency of the protective *ε2* allele is nearly two-fold increased (16% *vs* 9%) (Tesi et al., 2019). Note, however, that inclusion criteria of the centenarian cohort required them to self-report to be cognitively healthy, which might have increased the observed longevity effect. Apart from the *APOE* variants, the AD-group included 15 variants, all of which were among the first to be associated with AD through GWAS (in/near *CR1, CD33, BIN1, MS4A6A, PICALM,* and *SLC24A4*), eventually representing variants with the strongest effect on AD (Harold et al., 2009; Lambert et al., 2009). Functional annotation showed significant enrichment of β -amyloid metabolism, which aligns with the importance of functional Amyloid metabolism in maintaining brain health. We also observed functional enrichment of endocytosis and immune signaling, and a specific cell-type enrichment for microglia. This is in line with the currently growing hypothesis of the involvement of immune dysfunctions in the etiology of AD (Efthymiou and Goate, 2017; Hansen et al., 2018). Apart from *APOE*-associated SNPs, for which the effects on several neurological diseases, cardiovascular diseases as well as diabetes are well known (Liu et al., 2019), the other SNPs in this group were not associated with age-related diseases, although their associated genes have been linked to cardiovascular diseases (*PLCG2*, *ACE*, *FERMT2*) (Hoffmann et al., 2017; Koyama et al., 2020), cancer (*FERMT2*), (Conti et al., 2021), and diabetes (*ACE*). (Vujkovic et al., 2020).

#### Variants With a Larger Effect on Longevity Than Alzheimer’s Disease

The second-largest group of variants constituted a subset of 11 variants with a larger effect on longevity than the effect on AD, which suggests that these variants may be involved in other age-related diseases or general age-related processes. The AD-association of most of these variants is relatively recent, likely due to small effect sizes (ORs) or variants rareness; both features necessitate a very large number of samples to identify these variants as significantly associating with the disease. The variants within this group were specifically enriched for immune response and endocytosis, which are known hallmarks of longevity (Solé-Domènech et al., 2016; Partridge et al., 2018; Sadighi Akha, 2018). In addition to the low frequency, non-synonymous variant in the *PLCG2* gene (*rs72824905,* MAF: 0.6%), which was recently observed to be protective against AD, frontotemporal dementia (FTD) and dementia with Lewy bodies, other variants within this group were previously linked with disease risk factors. One of the two non-synonymous variants in the *SHARPIN* gene, variant *rs34173062* (MAF: 5.7%), has been associated with respiratory system diseases in GWAS (Astle et al., 2016; Kichaev et al., 2019; Olafsdottir et al., 2020). Variant *rs7185636* (MAF: 17.1%), intronic of the *IQCK* gene, is in complete linkage with a variant (*rs7191155*, *R* ^2^ = 0.95), which was previously associated with body-mass index (BMI) (Hoffmann et al., 2018). The variant *rs876461* (MAF: 13.0%) near the *PRKD3* gene is in linkage with variant *rs13420463* (*R* ^2^ = 0.42), which has been associated with systolic blood pressure (Warren et al., 2017). Further, the variant near *CD2AP* gene associates with the development and maintenance of the blood-brain barrier, a specialized vascular structure of the central nervous system which, when disrupted, has been linked with epilepsy, stroke and AD (Cochran et al., 2015). Variant *rs9275152* (MAF: 10.4%) maps to the complex Human-Leukocyte-Antigen (HLA) region, which codes for cell-surface proteins responsible for the regulation of the adaptive immune system. In numerous GWAS, variants in the HLA region were associated with autoimmune diseases, cancer, and longevity (Bodis et al., 2018; Timmers et al., 2019). The AD-associated variant in this region (*rs9275152*) is also a risk variant for Parkinson’s disease (Bandres-Ciga et al., 2019). Finally, the genomic region surrounding the *SPI1* gene (in which variant *rs3740688* maps) has been previously associated with cognitive traits (intelligence, depression) (Davies et al., 2018) and, with lower evidence, with kidney disease and cancer (Pattaro et al., 2016; Michailidou et al., 2017). The remaining variants *rs56402156*, *rs7920721,* and *rs4351014* (in/near *EPHA1, ECHDC3,* and *HS3ST1*) have not been directly associated with other traits, although their associated genes were implicated in systemic lupus erythematosus (*HS3ST1*) and cancer (*EPHA1, ECHDC3*) (Herath et al., 2009; Rafiq et al., 2014; Langefeld et al., 2017). Together, these findings suggest that the counterpart of each risk-increasing allele, the AD-protective alleles, might give a survival advantage that is not only specific to AD. Their functional and cell-type annotations suggest that they contribute to the maintenance of regulatory stimuli in the immune and endosomal systems, which may be essential to maintain brain and overall physical health, necessary to reach extremely old ages in good cognitive health (Tesi et al., 2020).

#### Variants Associated With Increased Risk of Alzheimer’s Disease and Increased Longevity Risk

Unexpectedly, ten variants increased the risk of AD while at the same time increasing the chance to reach ages over 100 in good cognitive health, which is an *unexpected* balance. We note that the IED distributions of these variants were broad, and in some cases even showed a bimodal behavior (in/near *KANSL1, IL34, CHRNE*): this is attributable to the small effect-sizes (and large standard errors) on longevity for these variants, which caused data points to easily flip between the expected and unexpected direction during the sampling procedure. Replication of the direction of the variant effect in an independent dataset of parental longevity indicated that the unexpected direction was replicated in only the *CLU* and *CHRNE* variants, suggesting that future studies will have to further explore (the robustness of) these unexpected effects. However, such unexpected effects are not new: for example, in a targeted study of SNPs in *IPMK* and *IP6K3* genes, authors showed that a SNP in *IP6K3* increased both the risk of late-onset AD and longevity, while SNPs in *IPMK* and *UCP4* genes were associated with a lower risk of both late-onset AD and longevity (Dato et al., 2021). In fact, one explanation for such counter-intuitive effects may be a variant interaction with other variants, also known as epistasis: this phenomenon may lead to the modification of the magnitude of the effect observed for a given SNP, and may even determine a change in the direction of the effect (Mackay, 2014; Mackay and Moore, 2014; Cole et al., 2017). Such interaction mechanisms were shown to occur in AD for the variant in the *KANSL1* and *CLU* gene with respect to the *APOE* genotype (Jun et al., 2016). Specifically, carrying the risk allele of such variants may specifically affect the risk of AD in *APOE ε4* allele carriers, which are not prevalent among cognitively healthy centenarians. It is thus possible that 1) the increased risk of AD conferred by these SNPs is mitigated by other SNPs in the genome, but also that 2) different forms of SNP-SNP interactions may have different effects on AD and longevity depending on the genetic architecture of these traits and their associated molecular pathways. An alternative explanation may be that these variants have age-dependent effects: for example, high blood pressure at midlife increases the risk of AD, but after the age of 85 a high blood pressure protects against AD (McGrath et al., 2017). Similarly, a high body-mass index (BMI) increases the risk of AD at midlife, while being protective at older ages (Xu et al., 2011). In line with this hypothesis, genes *PTK2B*, *KANSL1* and *ADAM10* have been previously associated with obesity and BMI (Hoffmann et al., 2018; Kichaev et al., 2019; Christakoudi et al., 2021), while *ABCA7* and *ADAM10* have been associated with blood pressure (Surendran et al., 2020; Bone et al., 2021). In addition, the AD variant in/near *IL34* gene codes for a cytokine that is crucial for the differentiation and the maintenance of microglia (Wang and Colonna, 2014): although further studies are needed, an excessive differentiation in middle-age individuals may increase brain-related inflammation and AD-risk, while it might compensate for the slower differentiation and immune activity at very old ages. Next to *IL34*, several genes may be affected by these Unex-variants, such as *PTK2B* and *INPP5D,* which play a role in aging-associated processes such as cellular senescence and immunity (Ryu et al., 2006; Pauls and Marshall, 2017).

### Strengths and Weaknesses

We acknowledge that our findings are based on relatively small sample sizes, especially for the cognitively healthy centenarian group. This phenotype is rare, and individuals need to be individually approached for study inclusion (Holstege et al., 2018), which is prohibitive for large sample collection. For this reason, we did not perform extensive gender-specific analyses and thus we cannot rule out possible interaction effects in males and/or females. However, we note that the frequency of AD-associated SNPs was not different between male centenarians and female centenarians. As population subjects in our comparison, we used individuals from five different cohorts: all individuals were from the same (Dutch) population, all tested cognitively intact, and did not develop dementia at the time of analyses. Although using a genetically homogeneous population represents a strength of this study, observed effects may differ in different populations and ethnicities, especially regarding the effects on longevity. This will require further study. It is known that the analysis of variants with small effect-sizes in relatively small sample sizes leads to large confidence intervals: we took this uncertainty into account by bootstrapping effect-sizes, causing the, IED distributions of several variants to be widely spread. By focusing our analysis on SNPs that were genome-wide significantly associated with AD (thus having tight confidence intervals), we limited this dispersion to the effects-sizes on longevity only. For this reason, we anticipate that using a random set of SNPs (*e.g.,* to investigate the basic properties of the IED ), would increase further more the dispersion of data points along the longevity-AD-unexpected spectrum, as confidence intervals on both longevity and AD would likely be larger. Although our work represents a first step towards understanding the effect of AD-associated variants on longevity, a replication analysis in larger cohorts of centenarians and/or long-lived individuals is warranted to further support our findings. Secondly, in the functional annotation analysis, we had to deal with the problem that the downstream effect of AD-associated variants is often unclear. To accommodate this uncertainty, we allowed multiple genes to be associated with each variant. However, it is likely that our variant-pathways annotation will change as we gain more understanding about these variant-effects, the likely affected genes, and their functions. When we inspected the parental-longevity GWAS, most of the variants that were in the *expected direction* in our study were also in the same direction in the GWAS; however, this was not true for all variants. The variant that deviated the most between our study and the parental-longevity GWAS was *rs9275152* in the *HLA* region: while we clustered this variant in the Longevity-group, in the parental-longevity GWAS the direction of effect was opposite (*i.e., unexpected*), suggesting that the variant increased the risk of AD and at the same time the odds of longevity (Timmers et al., 2019). The genomic region to which *HLA* maps is biologically known to be affected by many recombination events and may be population- and environment-dependent, which may explain this divergence (Jinam and Saitou, 2017). In addition to *HLA*-variant, variant *rs34674752* in the *SHARPIN* gene reported the second-largest effect-size in our study (after *APOE-ε4*), while the effect-size of this variant in the GWAS was very small, yet in the *expected direction.* To this end, we note that the individuals used in the parental-longevity GWAS were themselves not extremely old individuals, such that possible pleiotropic effects at very old ages, as described earlier, may not be observable in this GWAS. However, while we observed overall consistency in effect-size direction for variants in the expected direction, 6/8 of the variants in the unexpected direction were in the expected direction in the GWAS, with variants near *SORL1, IL34,* and *ADAM10* having the most noticeable differences. We speculate that the relatively young ages of the GWAS samples, together with the small sample size of our centenarian cohort may be the cause of such discrepancy.

## Conclusion

Most AD-associated variants that increase the risk of the disease are associated with lower odds of longevity. We identified a subset of variants with a larger effect on longevity than on AD, that were previously associated as risk-factors for other age-related diseases, and that are selectively enriched for endocytosis and immune signaling functions.

## Data Availability

The datasets presented in this study can be found in online repositories. The names of the repository/repositories and accession number(s) can be found in the article/Supplementary Material.
