# Supplementary material for: The Effect of Alzheimer’s Disease-Associated Genetic Variants on Longevity
Source: Front Genet. 2021 Dec 21;12:748781. doi: 10.3389/fgene.2021.748781 (PMC8724252; doi:10.3389/fgene.2021.748781)
Supplement: Supplementary file 1 [file DataSheet1.pdf]

Supplementary Material

1 Supplementary Methods

1.1 Populations

The 100-plus Study focuses on the biomolecular aspect of preserved cognitive health until extremely old ages. This study includes (1) Dutch-speaking centenarians who can (2) provide official evidence for being aged 100 years or older, (3) self-report to be cognitively healthy, which is confirmed by an informant (*i.e.* a child or close relation), (4) consent to donation of a blood sample and (5) consent to (at least) two home-visits from a researcher, which includes an interview and neuropsychological testing.[14] This study also includes (1) siblings or children from centenarians who participate in the 100-plus Study, or partners thereof who (2) agree to donate a blood sample, (3) agree to fill in a family history, lifestyle history, and disease history questionnaire. The Longitudinal Aging Study of Amsterdam (LASA) is an ongoing longitudinal study of older adults initiated in 1991, with the main objective to determine predictors and consequences of aging.[18, 19] The SCIENCe is a prospective cohort study of subjective cognitive decline (SCD) patients.[20, 21] Participants undergo extensive assessment, including cerebrospinal fluid collection (CSF) and optional amyloid positron emission tomography scan (PET), with annual follow-up. The primary outcome measure is clinical progression. All individuals were labeled cognitively intact. The Netherlands Brain Bank (NBB) cohort is a prospective donor program for psychiatric diseases. All subjects were labeled cognitively intact after neuropathological examination.[22] The Netherland Twin Registry study (NTR) was established in 2004 to collect biological and environmental data in twin families to create a resource for genetic studies on health, lifestyle, and personality.[23]

1.2 Genotyping and imputation

Genetic variants in our populations were determined by standard genotyping and imputation methods, and we applied established quality control methods: we genotyped all individuals with the Illumina Global Screening Array (GSAsharedCUSTOM_20018389_A2) and excluded individuals with low-quality genotypes (individual call rate <98%, variant call rate <98%), individuals with sex mismatches and variants deviating from Hardy-Weinberg equilibrium ($p<1x10^{-6}$). Genotypes were prepared for imputation comparing variants identifiers, strand and allele frequencies to the Haplotype Reference Panel (HRC v1.1, April 2016), and all remaining variants were submitted to the Sanger imputation server (https://imputation.sanger.ac.uk).[53] The server uses EAGLE2 (v2.0.5) to phase the data, and imputation to the reference panel was performed with PBWT.[54, 55] Before analysis, we excluded individuals of non-European ancestry

and individuals with a family relation, leaving 2,905 population subjects and 343 cognitively healthy centenarians for the analysis.

1.3 Variant annotation

1.3.1 Variant-gene mapping

We annotated each variant to the likely affected gene(s), so-called *variant-gene mapping*, combining annotation from *Combined Annotation Dependent Depletion* (CADD, v1.3), *expression-quantitative-trait-loci* in the blood (eQTL from GTEx v8), and positional mapping (from RefSeq build 98).[56-58] In the case of coding variants, we confidently associated the variant with the corresponding gene. Alternatively, we first considered possible eQTL associations. When these were not available, we included all genes at increasing distance d from the variant (starting with $d \leq 50kb$, up to $d \leq 500kb$, increasing by $50kb$ until at least 1 gene was found). Our procedure allows the association of each variant with one or multiple genes (*Figure S2*).

1.3.2 Gene-pathway mapping

The resulting list of genes was used to find the molecular pathways enriched in the AD variants. See *Figure S2* for a schematic representation of our annotation framework. We realized that allowing multiple genes to associate with each variant could result in an enrichment bias, as neighboring genes are often functionally related. To control this, we implemented a sampling technique: at each iteration, we (i) sampled one gene from the pool of genes associated with each variant, and (ii) performed a gene-set enrichment analysis with the resulting list of genes. The gene-set enrichment analysis was performed considering biological processes (BP) and implemented with the *enrichGO* function of the R package *clusterProfiler*, with all genes as background and correcting p-values controlling the False Discovery Rate (FDR). Finally, we averaged p-values for each enriched term over the iterations (N=1,000). To facilitate interpretation, we merged significantly enriched biological processes. First, we calculated the semantic similarity between all significant biological processes (*i.e.* FDR<5%) using *Lin* as a distance measure.[59] We then applied hierarchical clustering on the resulting distance matrix and selected the number of functional clusters using the dynamic tree-cut method as implemented in *cutreeDynamic* function from the R package *WGCNA*, specifying 15 as the minimum number of terms per cluster (using the default value of 20 resulted in 2 functional clusters only). To provide an interpretation of each functional cluster, we selected the most frequent words describing the biological processes underlying each cluster, and show this as word-clouds as implemented in R package *wordcloud2*. Finally, by counting how often a functional cluster was associated with a gene, we could calculate a weighted annotation of each gene to the 4 functional clusters, so-called

gene-pathway mapping (*Figure S2*). The variant-gene mapping as well as the gene-pathway mapping procedures were performed using the web-server application available at https://snpxplorer.net.[24] Due to the initial selection of significantly enriched BP, not every gene in the list of variant-associated genes is annotated with (at least one of) these terms. Consequently, these genes could not be related to the final functional clusters. To overcome this, we connect these genes to the functional clusters using a k-nearest neighbor (k-NN) imputation. The k-NN model was initially trained using the functional clusters as classes and the semantic similarity matrix between the enriched biological processes as features (*feature terms*). Then, for each gene with missing annotation, we (*i*) extracted all the biological processes the gene is involved in (*input biological processes*), and (*ii*) calculated the semantic similarity matrix between these terms and the *feature terms*, which defines the similarity between the *input biological processes* and the *feature terms*. Finally, we (*iii*) predicted the probability of classification of the similarity matrix to the classes (functional clusters), and used this as weight for the gene-pathway mapping (*Figure S2*).

1.3.3 Variant-pathway mapping

The variant-pathway mapping represents the combined annotation of each variant to the different functional clusters. As such, it depends on the variant-gene mapping and the gene-pathways mapping. Briefly, given a variant k, we (*i*) retrieved all the genes that were associated with the variant in the variant-gene mapping, G_k, and (*ii*) retrieved all the biological processes (gene ontology term identifiers) that were associated with these genes, GO_G. Because we clustered biological processes into functional clusters, by looking at which functional clusters the GO_G belonged to, we could assign a weight of association for variant k to each of the functional clusters.

1.4 Variant-cell-type mapping

To study brain-specific cell-types and their relationship with AD-associated variants, we used the publicly available gene expression dataset GSE73721: this dataset includes gene expression values of 6 fetal astrocyte samples, 12 adult astrocyte samples, 8 sclerotic hippocampal samples, 4 whole human cortex samples, 4 adult mouse astrocyte samples, and 11 human samples of other purified central-nervous-system (CNS) cell-types. We restricted to the gene expression of 12 astrocyte samples and 11 samples of purified CNS cell-types from the cortex of adult humans (total N=23, mean age of 41.5±19.6 years). To calculate the variant-cell-type mapping, we averaged the gene expression of the genes mapping to the same variant.

1.1 Supplementary Figures

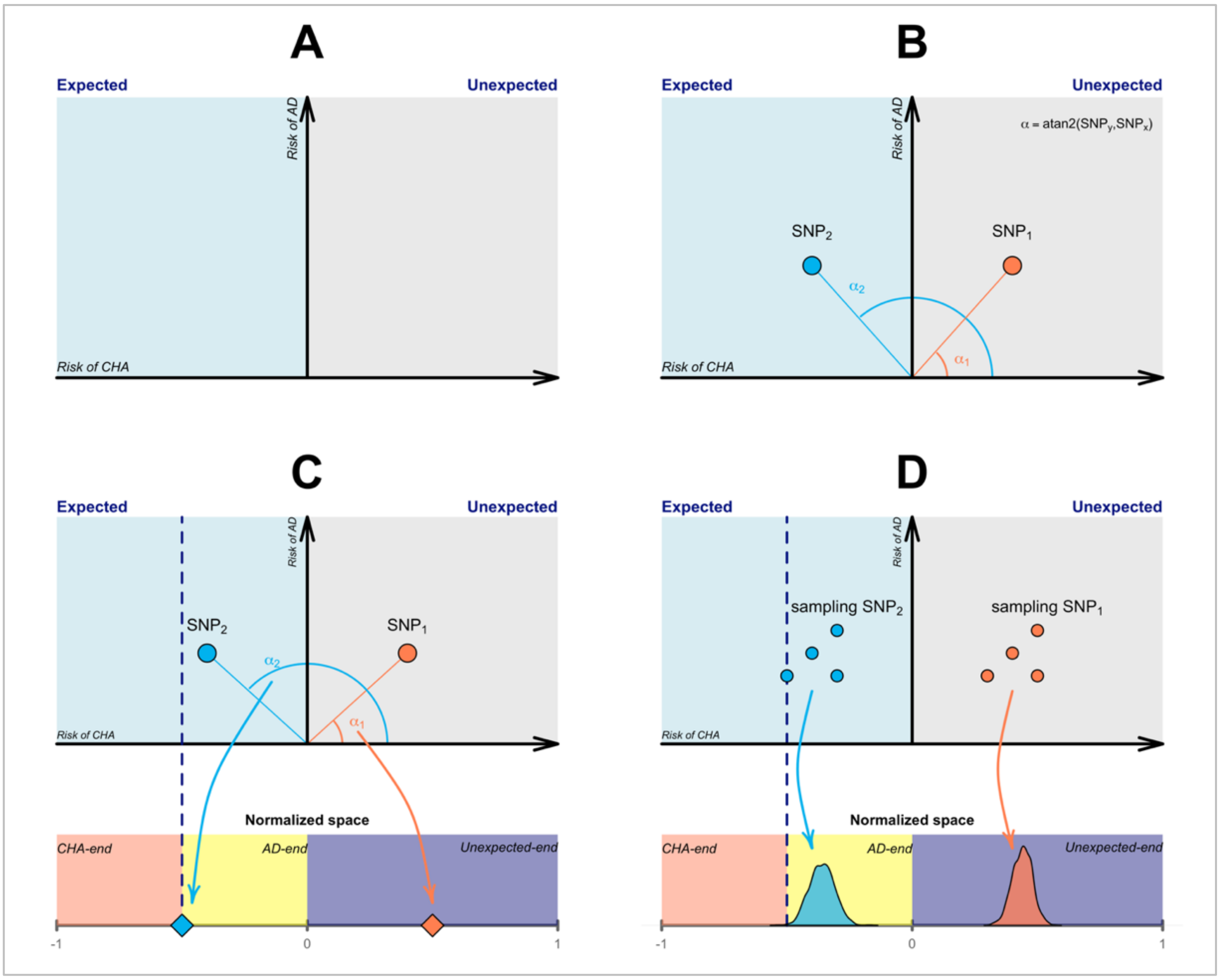

Figure S1: Explanation of the distribution of imbalance variant effect direction (IED). The figure shows the sequential steps for constructing the distribution of the expected direction of variant effect for AD-risk compared to longevity for two toy variants (SNP_1 and SNP_2). **A.** Axes definition, with the y-axis being the effect-size for AD-risk (log of odds ratio) of a variant, derived from literature and set positive by definition. The x-axis identifies the effect-size of a variant on longevity. This can be either positive or negative depending on the variant's association in cognitively healthy centenarians as opposed to population subjects. The blue area represents that the two effects are in the expected direction with respect to each other, *i.e.* a variant increases the risk of AD and at the same time decreases the chance of longevity. Oppositely, the grey area refers to the unexpected direction of effect. **B.** Two toy variants (SNP_1 and SNP_2) are shown as data points. α_{1-2} represents the angle of the data point vector with the x-axis. **C.** Normalization of the α_{1-2} value into an arbitrary space. Here, we used $[-1; 1]$. **D.** Repeating this procedure for each bootstrap iteration of each variant, we obtained the distribution of imbalance effect direction for each variant (IED). Values smaller than 0 indicate the *expected direction* of effect, whereas values larger than 0 refer to the *unexpected direction* of effects. Additionally, values close to

0 indicate a larger AD effect than longevity effect, and values close to -1 suggest that the variant's longevity effect is larger than the AD effect.

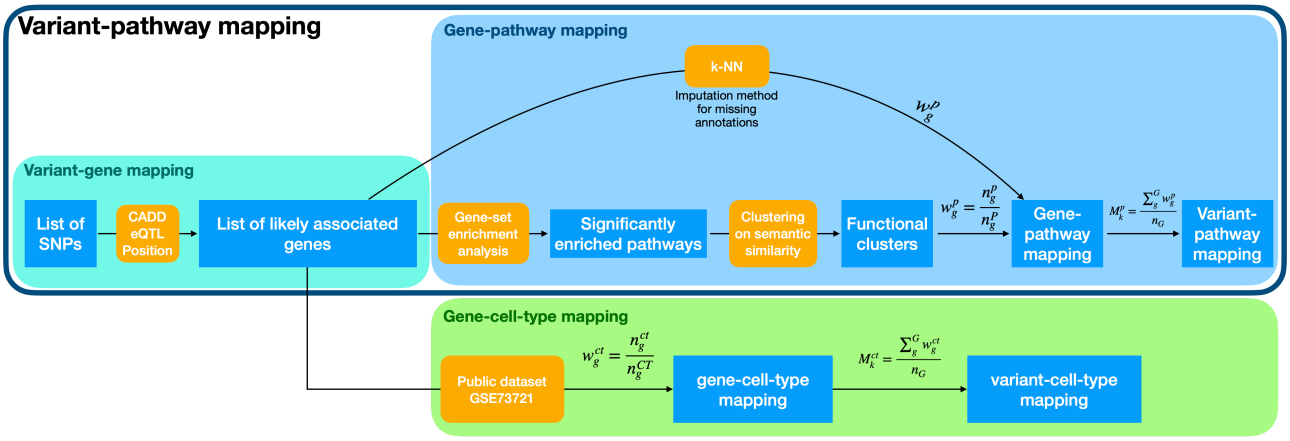

Figure S2: Schematic representation of the variant-pathway and variant-cell-type mapping. The figure shows a schematic representation of the annotation framework used to functionally annotate AD-associated variants and perform cell-type enrichment. Outputs are represented as blue squares, while methods are represented in orange. In the variant-gene mapping, showed in the grey box, we start from a list of variants and, through the integration of predicted variant consequences (CADD), eQTL and position, we obtain a list of genes. Note that here multiple genes may be associated with each variant. The yellow box shows the gene-pathway mapping: briefly, we perform gene-set enrichment analysis followed by clustering of the significantly enriched pathways to obtain functional clusters. We then calculate the gene-pathway mapping by looking at the (enriched) pathways associated with each gene and their associated functional clusters to get a weight for each gene-functional cluster association. Finally, we average the gene-pathway mapping of each gene associated with the same variant. Imputation methods (k-NN) are implemented for genes with missing annotation to obtain the gene-pathway mapping. Together, the grey box and the yellow box form the variant-pathway mapping. At the bottom, the green box shows the gene-cell-type enrichment using the public dataset GSE73721 of gene expression in different brain cell-types. Similar to the gene-pathway mapping, we calculate a weight of association of each gene to each cell-type, and we average these weights in case multiple genes mapped to the same variant (variant-cell-type mapping).

A

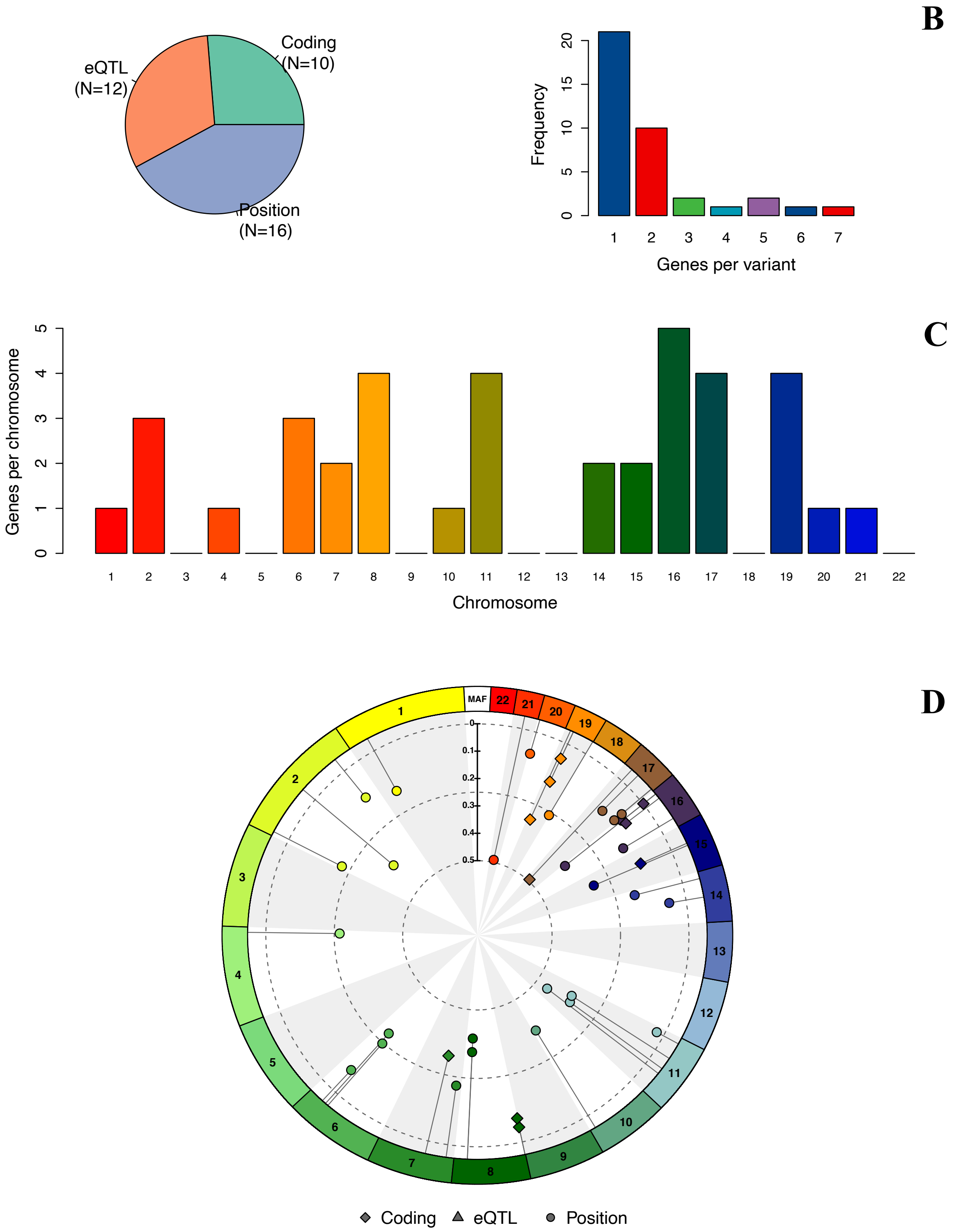

Figure S3: Variant-gene mapping for the 38 AD-associated variants. A. The sources used to annotate each variant to the likely affected genes. Coding: variants located in the coding region of a gene (*e.g.* synonymous or non-synonymous variants). eQTL: variants associated with RNA expression changes in blood from the GTEx consortium. *Position*: variants intronic or intergenic without evidence of eQTL associations that were annotated based on neighboring genes. **B.** Barplot

of the number of genes associated with each variant. **C.** Distribution of genes across the chromosomes. **D.** Distribution of the previously identified variants along the genome together with each variant's minor allele frequency and annotation.

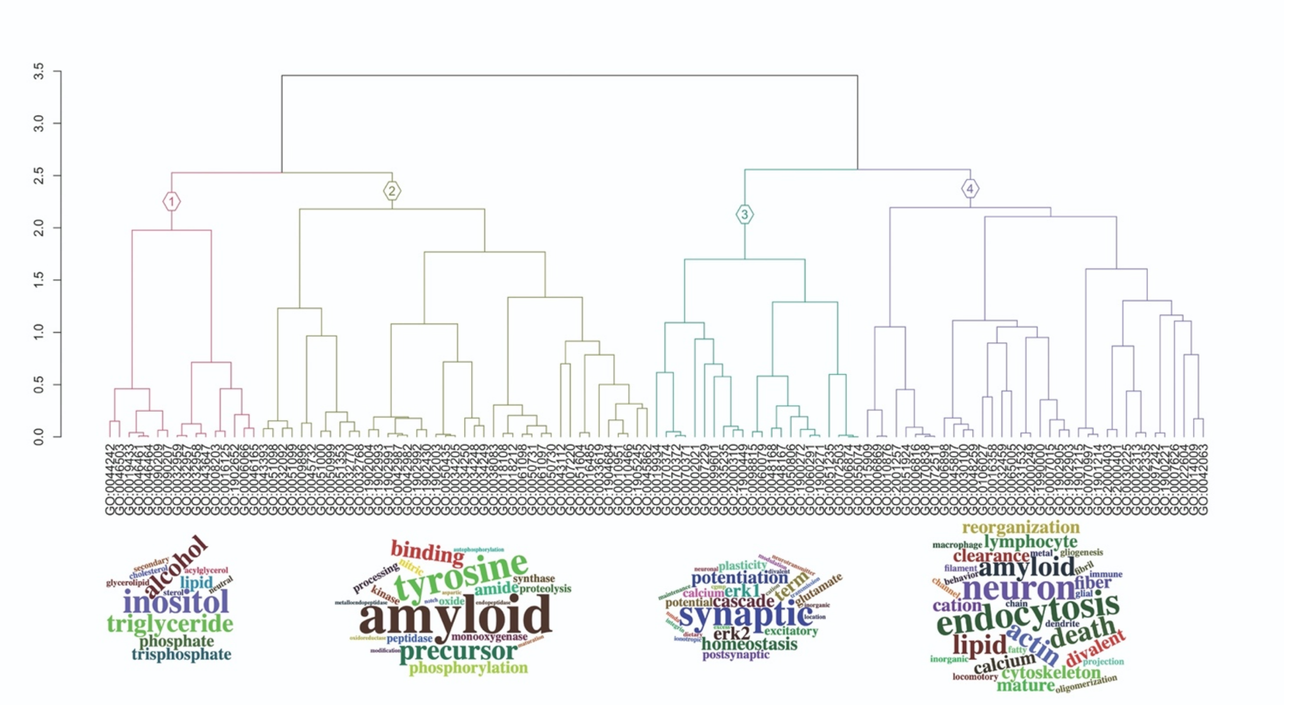

Figure S4: Hierarchical clustering of the semantic similarity matrix and the 4 functional clusters' definition. Dendrogram of the hierarchical clustering analysis and the 4 functional clusters, along with word-clouds of the most frequent terms per cluster. Hierarchical clustering was performed on the semantic similarity distance matrix (using *Lin* as semantic similarity metric). We used the dynamic tree-cut method to define the number of functional clusters, specifying 15 as the minimum number of terms per cluster. We then used word-cloud visualization as well as manual interpretation of the biological processes underlying each functional cluster to label each cluster to *Lipid/Cholesterol metabolism* (cluster 1), *β-Amyloid metabolism* (cluster 2), *Synaptic plasticity* (cluster 3) and *Endocytosis/Immune signaling* (cluster 4).
